# p53- and p73-independent activation of TIGAR expression *in vivo*

**DOI:** 10.1038/cddis.2015.205

**Published:** 2015-08-06

**Authors:** P Lee, A K Hock, K H Vousden, E C Cheung

**Affiliations:** 1 Cancer Research-UK Beatson Institute, Switchback Road, Glasgow G61 1BD, UK

## Abstract

TIGAR (*TP53*-induced glycolysis and apoptosis regulator) functions as a fructose-2,6-bisphosphatase and its expression results in a dampening of the glycolytic pathway, while increasing antioxidant capacity by increasing NADPH and GSH levels. In addition to being a p53 target, p53-independent expression of TIGAR is also seen in many human cancer cell lines that lack wild-type p53. Although human TIGAR expression can be induced by p53, TAp63 and TAp73, mouse TIGAR is less responsive to the p53 family members and basal levels of TIGAR expression does not depend on p53 or TAp73 expression in most mouse tissues *in vivo*. Although mouse TIGAR expression is clearly induced in the intestines of mice following DNA-damaging stress such as ionising radiation, this is also not dependent on p53 or TAp73.

TIGAR (*TP53*-induced glycolysis and apoptosis regulator) is a metabolic enzyme sharing structural similarities to the FBPase-2 domain of phosphofructokinase-2/fructose-2,6-bisphosphatase. TIGAR can act to lower the levels of fructose-2,6-bisphosphate (F-2,6-P_2_), an allosteric activator of phosphofructokinase-1 (PFK-1) in the glycolytic pathway. Lowering F-2,6-P_2_ levels results in decreased PFK-1 activity, thereby decreasing flux through glycolysis and potentially allowing for the diversion of glycolytic metabolites to other pathways such as the pentose phosphate pathway or the hexosamine pathway.^1, 2^ Although the detailed effects of TIGAR expression on metabolism remain to be determined, it is clear that TIGAR functions in many cell systems to mediate antioxidant defence through an increase in NADPH and GSH.^3, 4, 5, 6, 7, 8^ TIGAR has also been found to act as a 2,3-bisphosphoglycerate phosphatase, which catalyses the conversion of 2,3-bisphosphoglycerate into 3-phosphoglycerate,^9^ although the physiological significance of this activity remains unclear.

TIGAR was identified in human cells as a transcriptional target of the tumour-suppressor protein p53. The human *TIGAR* possesses two p53-binding sites, human p53-binding site (hBS) 1 and hBS2, where hBS2 is the functional p53-binding site.^2^ In the mouse genome, *Tigar* shows a similar organisation with two potential p53-binding sites, mBS1 and mBS2, in a similar arrangement as human *TIGAR*.^10^ As a p53 target, TIGAR would be predicted to play a role in tumour suppression and the antioxidant functions of TIGAR would be consistent with a role in protecting from the acquisition of damage. However, TIGAR expression has been found to be elevated in a number of cancer models and tumour types^4, 11, 12^ through a mechanism that is not dependent on the maintenance of wild-type (WT) p53. Moreover, the expression of TIGAR in human breast cancer was found inversely correlated to the levels of p53.^13^ Taken together, these data suggest that TIGAR can function in a tumour suppressor pathway as part of a p53 response, but may also contribute to cancer development when TIGAR expression is deregulated and uncoupled from p53. In mouse models, loss of TIGAR has been shown to result in a decreased ability to regenerate damaged intestinal epithelium and a restraint on tumour development, both situations where ROS limitation is important.^11^ These results are consistent with the model that the expression of TIGAR may support tumour progression.

Little is known about p53-independent expression of TIGAR, although other transcription factors such as SP1 and CREB^14, 15^ have been implicated. Other members of the p53 family (p63 and p73) are able to activate promoters of p53 targets such as p21^16, 17^ and these p53 family proteins can also contribute to the regulation of metabolic gene expression. It is therefore possible that p63 and p73 can also regulate TIGAR expression.

To further understand the regulation of TIGAR, we investigate the differences in TIGAR regulation by p53 and its family members. Although both p53 and TAp73 showed activity in promoting the expression of both human and mouse TIGAR reporters in cells, we found that the activation of expression of mouse TIGAR in response to genotoxic stress is not dependent on p53 or TAp73.

## Results

### TIGAR expression is varied across tissues

**Figure 1:**
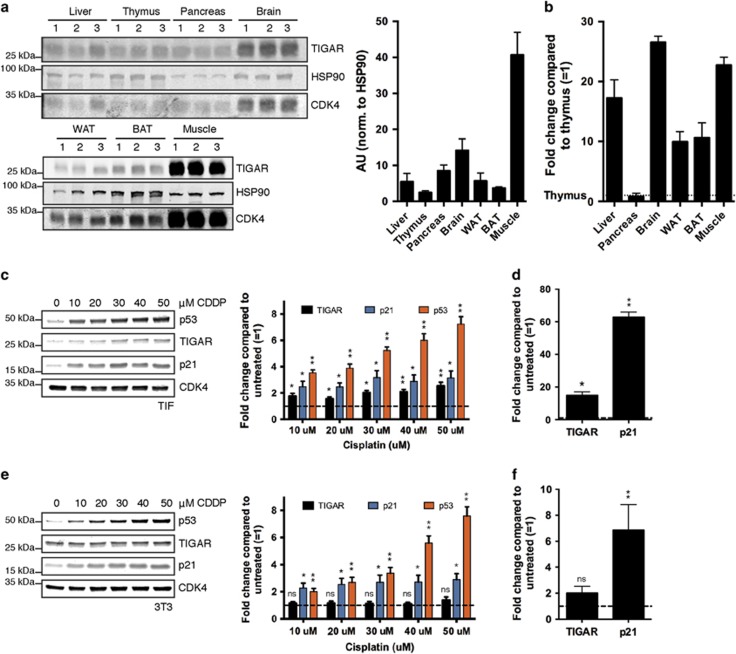
Basal TIGAR expression and its response to p53 activation. (**a**) Left: Western blot analysis of indicated tissues from three different wild-type (WT) animals. Right: Graph represents quantification of western blots. (**b**) mRNA expression of TIGAR in indicated tissues of WT animals. (**c**) Left: Western blot analysis of Tert-Immortalised Fibroblasts (TIFs) treated with indicated concentrations of cisplatin (CDDP) for 24 h. Right: Graph represents quantification of western blots with fold change compared with untreated. (**d**) mRNA expression of TIGAR and p21 following 24 h of CDDP treatment (50 *μ*M) in TIFs. (**e**) Left: Western blot analysis of 3T3s treated with indicated concentrations of CDDP for 24 h. Right: Graph represents quantification of western blots with fold change compared with untreated. (**f**) mRNA expression of TIGAR and p21 following 24 h of CDDP treatment (50 *μ*M) in 3T3s. Right: Graph represents quantification of western blots with fold change compared with untreated. Values represent mean±S.E.M. of three independent experiments unless otherwise indicated. *P<0.05, **P<0.005 compared with untreated unless otherwise indicated. NS, not significant

Although we have previously shown TIGAR to be expressed in several mouse tissues, to assess the relative levels of TIGAR expression, protein levels were evaluated across various tissues from WT mice (Figure 1a). TIGAR protein was detected in all tissues examined, with highest levels in the muscle and brain. Antibody specificity was confirmed using small intestine tissue from WT and TIGAR-deficient animals after treatment with irradiation (IR), which we have previously shown to increase TIGAR expression.^11^ As expected, TIGAR protein expression increased following IR in the WT animals and was not detected in TIGAR^−/−^ animals (Supplementary Figure 1a). Interestingly, TIGAR protein expression in tissues was not completely mirrored by mRNA expression (Figure 1b). For example, the protein expression of TIGAR in the liver and pancreas are similar, however, the levels of TIGAR mRNA in the pancreas are much lower than in the liver. This suggests additional mechanisms to regulate TIGAR protein levels may exist in some tissues.

### Mouse TIGAR is not responsive to p53 during genotoxic stress *in vitro*

Published studies have shown that mouse TIGAR can also be responsive to p53's transcriptional activity^10, 18, 19^ and p53-deficient mice lose the ability to induce TIGAR expression following myocardial injury.^19, 20^ However, TIGAR was also shown to be induced in mouse primary neurons following oxygen and glucose deprivation/reoxygenation in a p53-independent manner.^8^ To compare the p53-induced expression of TIGAR in mouse and human cells, we treated human tert-immortalised fibroblasts (TIFs) and mouse 3T3s with increasing concentrations of cisplatin (CDDP) to activate p53, but not induce cell death. After treatment, TIFs showed an increase in p53 protein level, along with an increased expression of TIGAR and a known p53 target, p21 (Figure 1c). However, although mouse 3T3s showed an elevation in p53 and p21, the expression of TIGAR was not detectably affected after treatment (Figure 1e). Similarly, using qRT-PCR to examine mRNA expression, human TIFs showed a significant increase in TIGAR mRNA expression after CDDP treatment that was not seen in the mouse cells (Figures 1d and f). These results suggest that p53 activation in mouse cells in culture does not consistently induce TIGAR expression.

### Loss of p53 does not affect expression of TIGAR *in vivo* following IR

**Figure 2:**
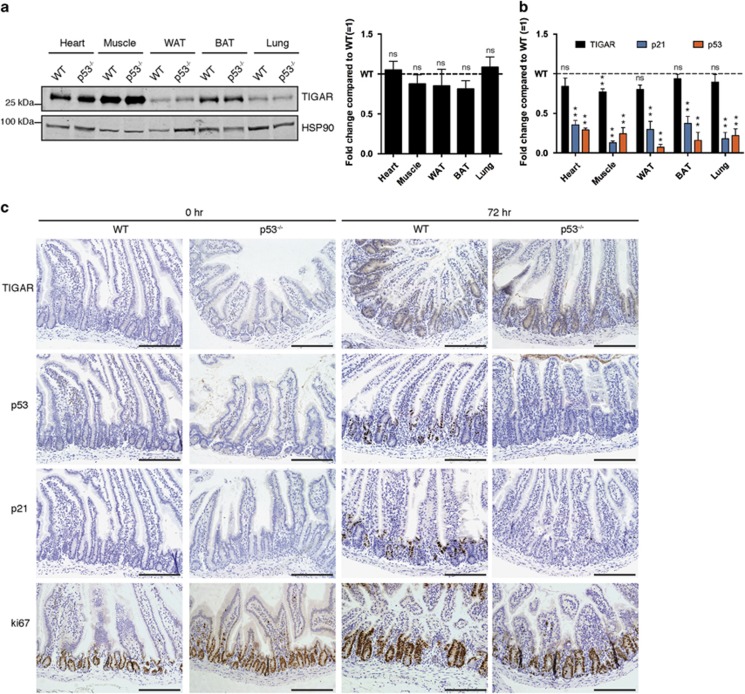
TIGAR expression in p53-null animals. (**a**) Left: Western blot analysis of TIGAR protein expression in organs of wild-type (WT) and p53^−/−^ mice. Right: Graph represents quantification of western blots with fold change compared with WT. (**b**) mRNA expression of TIGAR, p21 and p53 in organs of WT and p53^−/−^ mice. (**c**) Immunohistochemistry on small intestines from WT and p53^−/−^ animals 72 h after 10 Gy IR. Scale bar, 20 *μ*m. Values represent mean±S.E.M. of three independent experiments. *P<0.05, **P<0.005 compared with WT. NS, not significant. WAT, white adipose tissue. BAT, brown adipose tissue

Previous work has shown that TIGAR expression levels are increased in the crypts of WT mice during intestinal regeneration following tissue ablation.^11^ As p53 is also upregulated in the small intestine following IR,^21^ we examined whether TIGAR expression is controlled by p53 in mice *in vivo*. The basal TIGAR protein levels were examined in various organs of WT and p53^−/−^ mice. No significant reduction in TIGAR expression was seen in response to loss of p53 at either the protein level (Figure 2a) or the mRNA level (Figure 2b) – with a possible exception of a slight reduction in TIGAR mRNA in p53-null muscle. By contrast, p21 showed a very clear decrease in mRNA expression in all the p53^−/−^ organs examined.

To extend these studies, we tested whether a p53-dependent increase in TIGAR expression would occur *in vivo* after damage, focusing on the intestinal system in which we have previously shown increased TIGAR in response to IR. Antibody specificity for TIGAR immunohistochemistry was confirmed using small intestine tissue from WT and TIGAR-deficient animals after treatment with IR to induce TIGAR expression. As shown previously,^11^ TIGAR expression increased in the crypts of WT mice following IR, whereas no staining was observed in TIGAR^−/−^ animals (Supplementary Figure 1b). Comparison of WT and p53^−/−^ mice showed normal crypt architecture and similar levels of proliferation, as indicated by Ki67 staining, under unstressed conditions (Figure 2c). The basal expression of p53, p21 and TIGAR was also low in the crypts of WT and p53^−/−^ animals (Figure 2c). Tissue ablation of the intestinal epithelium by IR was followed by a period of recovery during which rapid tissue regeneration and proliferation occurred in WT and p53^−/−^ mice.^22^ Moreover, TIGAR expression increased in the crypts of both WT and p53^−/−^ animals, whereas p21 induction was clearly lower in the p53^−/−^ animals (Figure 2c). These data show that p53 is not necessary to maintain basal expression of TIGAR in many tissues or induce TIGAR expression following tissue damage in the small intestine.

### Comparison of human and mouse TIGAR p53-binding site activity

**Figure 3:**
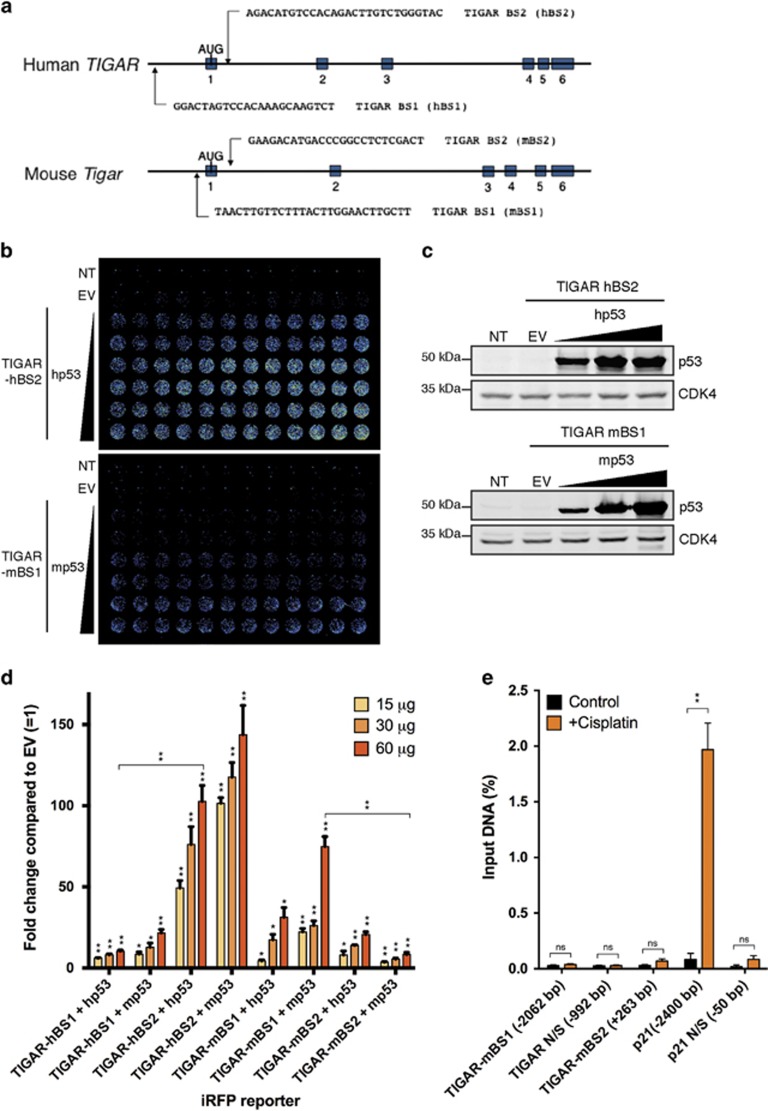
Comparison of human and mouse p53-binding sites on the *TIGAR* promoter. (**a**) Possible p53-binding sites along human and mouse *TIGAR*. (**b**) Representative iRFP reporter assay scan of HCT116 p53^−/−^ cells 24 h after co-transfection with TIGAR-hBS2 or TIGAR-mBS1 iRFP reporter and increasing amounts of human p53 or mouse p53. (**c**) Western blot analysis of HCT116 p53^−/−^ cells transfected with increasing amounts of human p53 or mouse p53. (**d**) Quantification of iRFP reporter scans on human (hBS1 and hBS2) and mouse (mBS1 and mBS2) TIGAR promoter-binding sites with increasing levels of human or mouse p53. (**e**) Chromatin-immunoprecipitation (ChIP) was performed for p53 with quantitative PCR for mBS1 (−2062 bp), mBS2 (+263 bp), a p53 response element on the *p21* promoter (−2400 bp) and non-specific (N/S) binding regions on the *Tigar* (−992 bp) and *p21* promoter (−50 bp), using 3T3s treated with 50 *μ*M cisplatin for 24 h. Values represent mean±S.E.M. of three independent experiments. *P<0.05, **P<0.005 compared with empty vector (EV) or control. NT, non-transfected; NS, not significant

The *in vitro* and *in vivo* data suggest that murine TIGAR is only weakly responsive to p53, possibly due to the differences in p53-binding sites between human and mouse TIGAR (Figure 3a). To investigate the differences between the human (hBS1 and hBS2) and mouse (mBS1 and mBS2) p53-binding sites of TIGAR directly, sequences corresponding to each p53-binding sites were cloned into infrared fluorescent protein (iRFP) reporter constructs.^23^ These constructs were co-transfected into HCT116 p53^−/−^ cells with increasing amounts of human or mouse p53 (Figures 3b and c). Each of these p53-binding site reporters were activated by both human and mouse p53. TIGAR-hBS2, the more efficient of the two human p53-binding sites, is efficiently activated by either human or mouse p53 (Figure 3d). By contrast, TIGAR-mBS1 is more responsive to p53 than TIGAR-mBS2, and slightly more responsive than TIGAR-hBS1, although less active than TIGAR-hBS2. Interestingly, mouse p53 was slightly more effective in the induction of all the binding site reporters, with the exception of TIGAR-mBS2. Taken together, the results suggest that the weaker p53-binding site (BS1) is structurally and functionally conserved between mouse and human but the stronger BS2 in humans is only very weakly active in the mouse.

To determine whether p53 can bind to either of the two putative binding sites in the mouse *Tigar* promoter, chromatin-immunoprecipitation was carried out in mouse 3T3 cells treated with CDDP to activate p53 (Figure 3e). Although p53 was clearly recruited to the p21 promoter following treatment, no increased binding of p53 to either mBS1 or mBS2 could be detected in these cells. The failure to recruit p53 to the *Tigar* promoter can explain the observed inefficiency of p53-dependent activation of mouse TIGAR expression seen in several cell types *in vitro* and *in vivo*.

### TAp73*α* can activate the human TIGAR p53-binding site reporter

**Figure 4:**
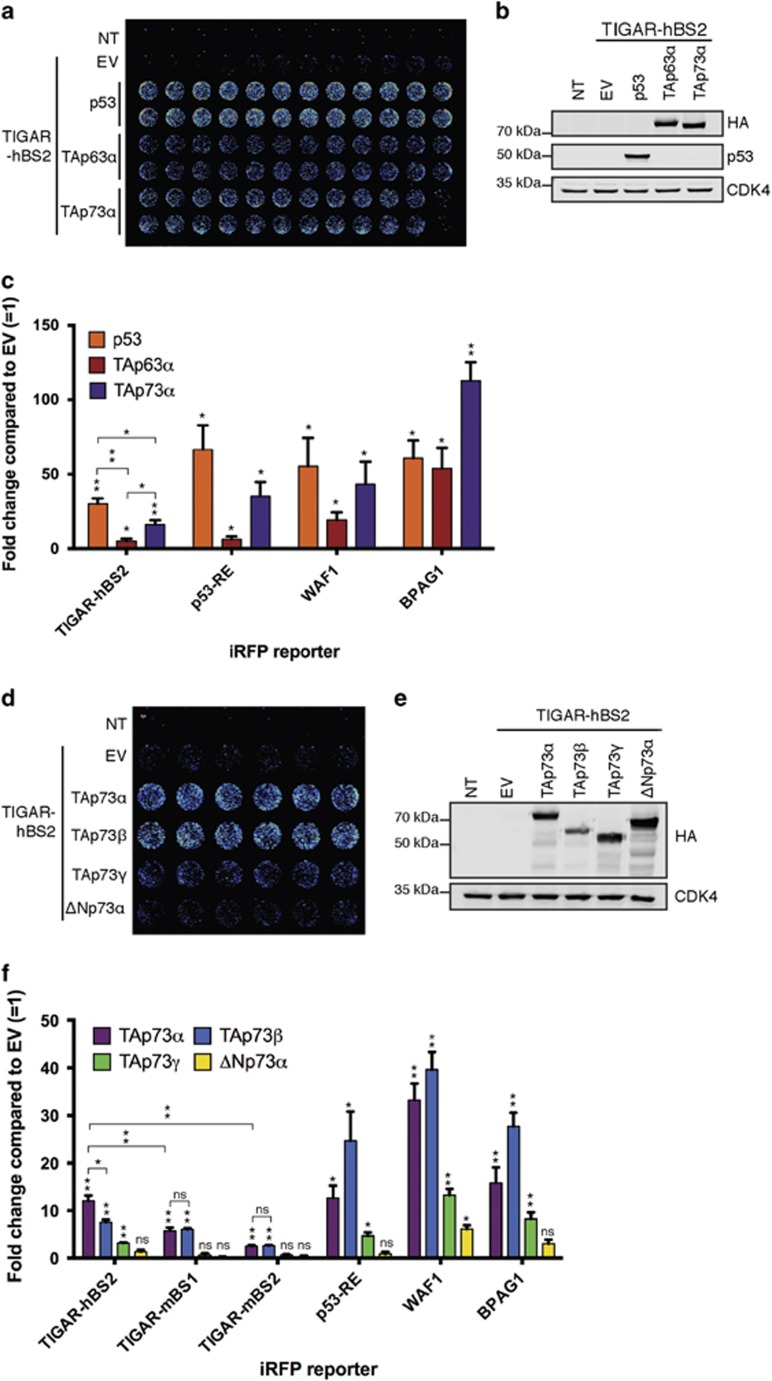
TAp63*α* and TAp73*α* can activate the TIGAR-hBS2 reporter. (**a**) Representative iRFP reporter assay scan of HCT116 p53^−/−^ cells 24 h after co-transfection with TIGAR-hBS2 iRFP reporter along with human p53, HA-tagged TAp63*α* or HA-tagged TAp73*α*. (**b**) Western blot analysis of HCT116 p53^−/−^ cells with transfected p53, HA-tagged TAp63*α* or HA-tagged TAp73*α*. (**c**) Quantification of iRFP reporter scans. (**d**) Representative iRFP reporter assay scan of HCT116 p53^−/−^ cells 24 h after co-transfection with TIGAR-hBS2 iRFP reporter along with TAp73*α*, TAp73*β*, TAp73*γ* or ΔNp73*α*. (**e**) Western blot analysis of HCT116 p53^−/−^ cells with transfected HA-tagged TAp73*α*, HA-tagged TAp73*β*, HA-tagged TAp73*γ* or HA-tagged ΔNp73*α*. (**f**) Quantification of iRFP reporter scans on human (hBS2) and mouse (mBS1 and mBS2) TIGAR promoter-binding sites. Values represent mean±S.E.M. of three independent experiments. *P<0.05, **P<0.005 compared with empty vector (EV). NT, non-transfected; NS, not significant

We further investigated the potential role of other p53 family members in the regulation of TIGAR expression. We first focused on the functional human p53-binding site (hBS2), co-transfecting the TIGAR-hBS2 iRFP reporter construct with p53, TAp63*α* or TAp73*α* to assess transcriptional activity. As positive controls we used iRFP expression constructs containing p53 response element encoding repeats of a known p53-binding sequence (p53RE), the p53-binding site of p21 (WAF1^24^) and a p63 response element from the skin-specific promoter of bullous pemphigoid antigen 1 (BPAG1^25^). Both TAp63*α* and TAp73*α* induced a response from the human TIGAR-hBS2 iRFP reporter construct, although the activity of TAp63*α* was extremely weak. The pattern of expression from TIGAR-hBS2 was similar to that seen with the p53RE or WAF1, where p53 was the most efficient, followed by TAp73*α*, then TAp63*α*. Strong activity for TAp63*α* was only measured using the BPAG1 promoter, although even here TAp73*α* was more active (Figures 4a–c). In light of these results, we focused on TAp73 isoforms as potential activators of TIGAR expression.

The TAp73*α* isoform has been shown to contain an inhibitory domain that limits its activity, making it less efficient than other isoforms.^26^ We therefore examined the activity of p73 isoforms, TAp73*α*, TAp73*β*, TAp73*γ* or ΔNp73*α*, in these assays. Although full-length TAp73 isoforms can induce p53 target genes,^27^ ΔNp73 isoforms, which lack the N-terminal activation domain,^28^ have been shown to inhibit TAp73 transcriptional activity as well as regulating an additional set of target genes.^29^ As expected,^30, 31^ TAp73*β* was consistently more effective in driving expression from p53RE, WAF1 or BPAG1 promoters. In these assays, TAp73*γ* and ΔNp73*α* did not show strong transcriptional activity. Turning to the reporter constructs containing TIGAR p53-binding sites (hBS2, mBS1 and mBS2), we found that TAp73*α* more effectively induced expression from hBS2, whereas both TAp73*α* and TAp73*β* modestly induced expression from mBS1 and mBS2 (Figures 4d–f). Taken together, the data suggest that like p53, TAp73 has the potential to drive the expression of both mouse and human TIGAR.

### Loss of TAp73 does not affect expression of TIGAR *in vivo* following IR

**Figure 5:**
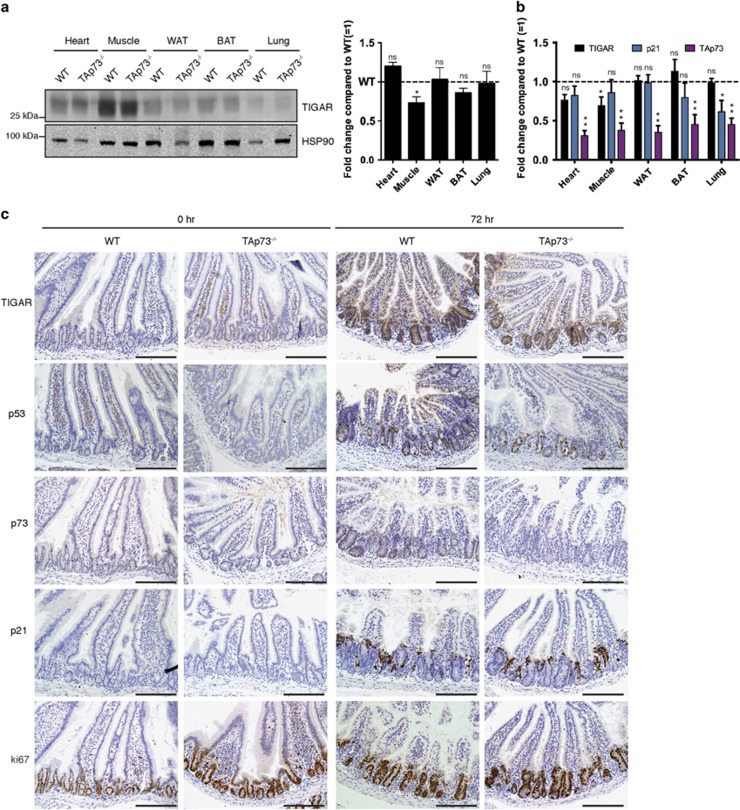
TIGAR expression in TAp73-null animals. (**a**) Left: Western blot analysis of TIGAR protein expression in organs of wild-type (WT) and TAp73^−/−^ mice. Right: Graph represents quantification of western blots with fold change compared with WT. (**b**) mRNA expression of TIGAR, p21 and TAp73 in organs of WT and TAp73^−/−^ mice. (**c**) Immunohistochemistry on small intestines from WT and TAp73^−/−^ animals 72 h after 10 Gy IR. Scale bar, 20 *μ*m. Values represent mean±S.E.M. of three independent experiments. *P<0.05, **P<0.005 compared with WT. NS, not significant; WAT, white adipose tissue; BAT, brown adipose tissue

p73 can be activated by DNA damage,^32, 33, 34^ potentially mediating the induction of TIGAR expression in response to IR independently of p53. To investigate this, we examined TIGAR expression in TAp73-deficient (TAp73^−/−^) mice. First, the basal expression of TIGAR was assessed in various organs of untreated WT and TAp73^−/−^ mice (Figure 5a). As seen in p53^−/−^ mice, no clear significant decrease in TIGAR expression was seen in TAp73^−/−^ tissues, with a possible small reduction in protein and mRNA levels in the muscle (Figure 5b). Following IR, intestines of TAp73^−/−^ mice underwent rapid proliferation, as shown by the proliferative marker Ki67 (Figure 5c). Although induction of TAp73 was limited to the WT mice, TIGAR expression was increased in the crypts of both WT and TAp73^−/−^ animals, showing that this induction of expression was not dependent on TAp73. p21 levels were also induced, reflecting the accumulation of p53 in response to IR in the TAp73^−/−^ animals (Figure 5c).

**Figure 6:**
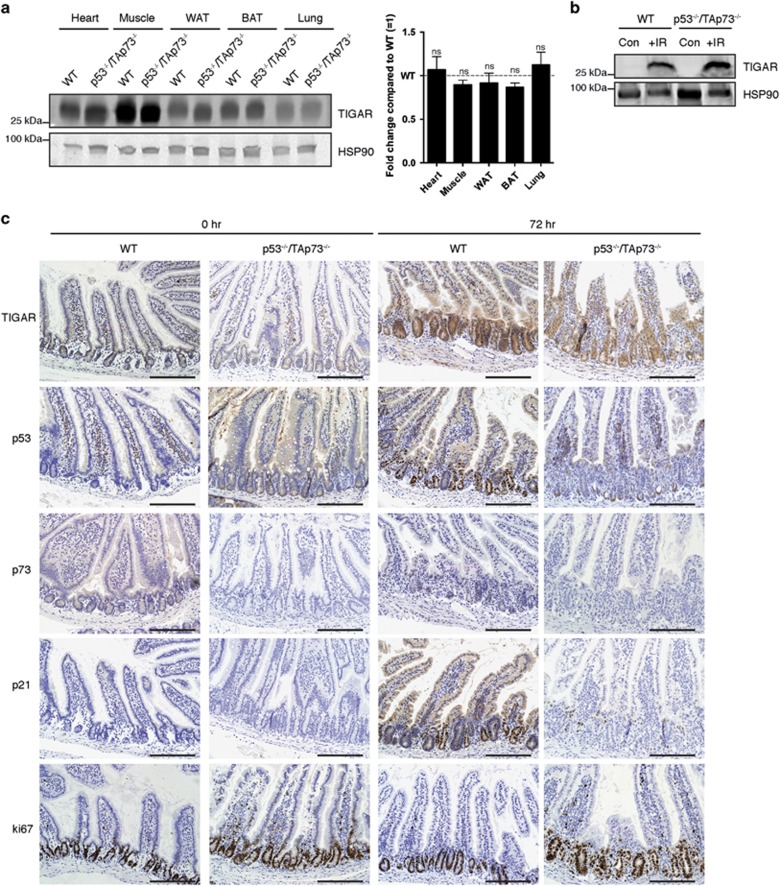
TIGAR expression in p53- and TAp73-null animals. (**a**) Left: Western blot analysis of TIGAR protein expression in organs of wild-type (WT) and p53^−/−^TAp73^−/−^ mice. Right: Graph represents quantification of western blots with fold change compared with WT. (**b**) Western blot analysis of TIGAR protein expression in small intestine tissue of WT and p53^−/−^TAp73^−/−^ mice 72 h after 10 Gy IR. Tissues were harvested from one experiment. (**c**) Immunohistochemistry on small intestines from WT and p53^−/−^TAp73^−/−^ animals 72 h after 10 Gy IR. Scale bar, 20 *μ*m. Values represent mean±S.E.M. of two independent experiments unless otherwise indicated. NS, not significant; WAT, white adipose tissue; BAT, brown adipose tissue

Finally, we examined possible redundancy between p53 and TAp73 in the induction of TIGAR expression after IR, by examining the effect of simultaneous deletion of both transcription factors. Compared with WT animals, there was no significant decrease in TIGAR expression in the organs of p53^−/−^TAp73^−/−^ mice (Figure 6a). Intestinal tissue from both WT and p53^−/−^TAp73^−/−^ animals showed a similar increase in TIGAR protein expression following IR that was detected by western blot of tissue samples or IHC of crypts (Figures 6b and c). Taken together, these data show that the increase in TIGAR expression seen following IR and gut regeneration is not dependent on p53 or TAp73 in mouse.

## Discussion

We have previously shown that TIGAR is induced following IR-induced intestinal damage and supports regeneration in the mouse. In humans, TIGAR is a p53 target gene and found to have a role in conditions of mild stress to promote cell survival.^2, 5^ We showed here that TAp73 can also activate expression from the TIGAR promoter in human cells. As IR can activate both p53 ^21^ and TAp73,^32, 33, 34^ we sought to test the hypothesis that the increase in TIGAR seen in mouse intestine following IR is a response to p53 and/or TAp73.

Our studies in cultured cells did not show a clear p53-dependent increase in TIGAR expression in mouse cells. A closer examination of the transcriptional control regions of human and mouse TIGAR showed that the principal p53-responsive element in human TIGAR is not well conserved in mouse TIGAR and is much less responsive to p53. The second, weaker binding site in humans seems to be conserved and somewhat more responsive to p53 in mouse. However, overall, the p53-binding sites in the human TIGAR promoter appear to be more responsive than those found in the mouse. TAp73 was also able to activate expression of mouse and human TIGAR-binding site reporters.

Despite the potential for both p53 and TAp73 to activate TIGAR expression, we found that although basal levels of TIGAR expression vary significantly between different mouse tissues, they are generally not affected by the loss of p53 or TAp73. Furthermore, the induction of TIGAR in mouse small intestine in response to IR does not depend on p53 or TAp73. Mice deficient for both p53 and TAp73 maintain a similar basal expression of TIGAR to WT animals and retain the ability to upregulate the expression of TIGAR in the crypts of the small intestine following tissue ablation. Importantly, several previous studies have shown p53-responsive expression of TIGAR in mouse cells and tissues such as the liver and heart, and p53 binding to the *Tigar* promoter was also detected in the liver.^10, 18, 19, 20^ We also found a significant, but minor, reduction in TIGAR expression in p53 or TAp73-deficient muscle (Figures 2 and 5). Taken together, the data suggest that although p53 can induce TIGAR in some mouse tissues, the p53-responsiveness of mouse TIGAR expression is lower than observed in human cells. To some extent this difference reflects the binding of p53 to the different response elements in the mouse and human TIGAR-encoding genes. However, it is also possible that tissue or stress-specific co-factors (that may show human/mouse differences in expression or availability) are required to allow p53 regulation of TIGAR expression. Given the function of TIGAR as a regulator of metabolism, it will be of particular interest to see whether p53 family proteins with other co-factors can participate in the induction of TIGAR in response to different forms of metabolic stress.

TIGAR has been found to be elevated in several human tumour types.^4, 11, 12^ The expression of TIGAR under these conditions does not correlate with the maintenance of WT p53,^13^ suggesting that TIGAR overexpression in tumours can be uncoupled from the activity of p53. Our data show that mouse TIGAR expression is also regulated through p53-independent mechanisms, and is strongly activated in intestinal crypts following IR and APC deletion.^11^ These observations suggest that activation of the Wnt signalling pathway may contribute to the regulation of TIGAR, particularly in the small intestine where this pathway has a key role in cell proliferation. Moreover, other transcription factors such as SP1 and CREB^14, 15^ have been shown to have a role in regulating the basal expression of TIGAR in liver cancer cell lines. Future studies will be required to establish how TIGAR expression is regulated during stress and whether deregulation of these pathways explains the elevated expression of TIGAR seen in human tumours.

## Materials and Methods

### Cell culture

All cell lines were cultured in Dulbecco's modified Eagle's medium (DMEM) supplemented with 10% of fetal bovine serum, 1% of glutamine, 1% of penicillin/streptomycin (Life Technologies, Paisley, UK), grown in a 37 °C incubator at 5% CO_2_. CDDP (Sigma-Aldrich, St. Louis, MO, USA) was used at the indicated concentrations and times.

### Small intestinal crypt culture

Small intestinal crypt culture was performed as previously described.^35^ Small intestine was washed in cold PBS and villi were scraped off using a glass coverslip. The small intestine was then cut into small pieces and further washed in cold PBS. This was then transferred into PBS containing 2 mM EDTA and incubated for 30 min. Crypts were then obtained via mechanical pipetting and the supernatant containing the crypts was collected. The crypts were centrifuged at a low speed (700 r.p.m., 3 min) to remove single cells and the final pellet was resuspended in growth factor reduced Matrigel (BD, Franklin Lakes, NJ, USA). Crypts were cultured in Advanced DMEM/F-12 (Life Technologies) supplemented with 1% of glutamine, 1% of penicillin/streptomycin, 0.1% of AlbuMAX I (Life Techologies), 10 mM HEPES (Life Technologies), 0.05 *μ*g/ml EGF (Peprotech, Rockyhill, NJ, USA), 0.1 *μ*g/ml Noggin (Peprotech) and 0.5 *μ*g/ml mR-spondin (R&D Systems, Minneapolis, MN, USA).

### Animals

All animal work was carried out in-line with the Animals (Scientific Procedures) Act 1986 and the EU Directive 2010 and sanctioned by Local Ethical Review Process (University of Glasgow). The p53^−/−^^36^ and TAp73^−/−^^37^ animals have been previously described.

### Western blot

Cell lysates were prepared in RIPA buffer with complete protease inhibitors (Roche, Penzberg, Germany), resolved via PAGE and transferred to nitrocellulose membranes. The following primary antibodies were used: Actin I-19- R (Santa Cruz Biotechnology, Dallas, TX, USA), cyclin D1 (Cell Signalling Technology, Danvers, MA, USA), CDK4 C-22 (Santa Cruz Biotechnology), HA.11 16B12 (Covance, Princeton, NJ, USA), HSP90 (Cell Signalling Technology), p21 C-19 (Santa Cruz Biotechnology), p53 1C12 (Cell Signalling Technology), p53 DO-1 (Santa Cruz Biotechnology), TIGAR G-2 (Santa Cruz Biotechnology) and TIGAR M-209 (Santa Cruz Biotechnology). Secondary antibodies were IRDye800CW-conjugated (LiCor Biosciences, Lincoln, NE, USA) and detection was performed using an Odyssey infrared scanner (LiCor Biosciences).

### Gene expression analyses

RNA was isolated from cells or mouse tissue using the RNeasy RNA Isolation kit according to the manufacturer's instructions (Qiagen, Valencia, CA, USA). Mouse TIGAR primer was purchased from Qiagen and mouse GAPDH was used as murine housekeeping gene (Primer Design, Southampton, UK).

mRNA primer sequences (5'→3'):


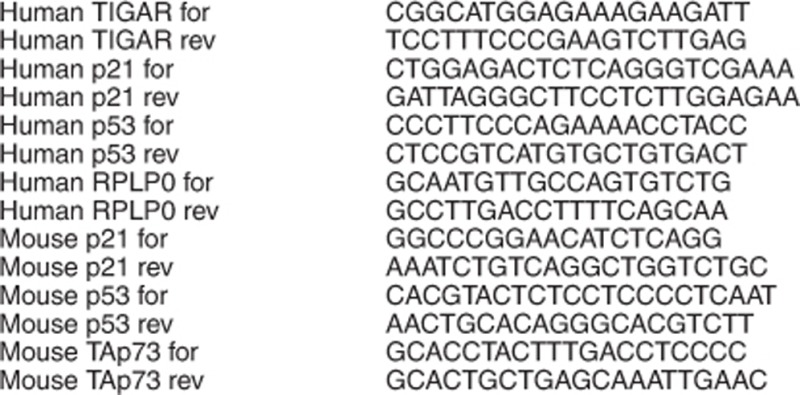


### IR treatment

Gamma IR-induced intestinal damage was performed as previously described.^38^

### Immunohistochemistry

Immunohistochemistry was performed as previously described.^39^ Primary antibodies used were: TIGAR (Merck Millipore, Darmstadt, Germany), p53 CM-5 (Vector Laboratories, Peterborough, UK), p21 M-19 (Santa Cruz Biotechnology), p73 S-20 (Santa Cruz Biotechnology) and Ki67 (Thermo Scientific, Waltham, MA, USA).

### Plasmids

pcDNA3.1+ (Invitrogen, Grand Island, NY, USA) was used as empty vector control. iRFP reporter constructs were generated as previously described.^23^ Reporter elements were ligated into vectors using the InFusion HD Eco Dry system (Clontech, Saint-Germain-en-Laye, France) according to the manufacturer's instructions.

Insert primer sequences (5'→3'):


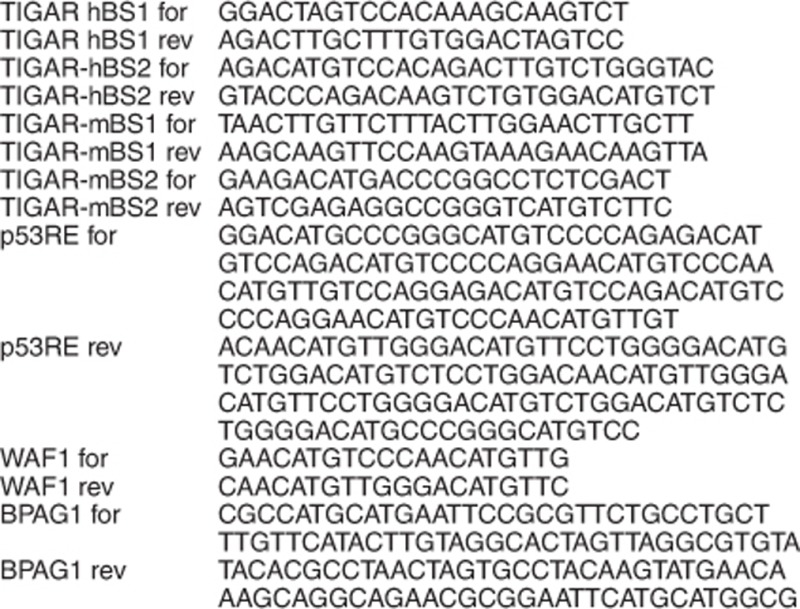


### Transient transfections and irfp reporter assays

Cells were seeded on 6-well plates for protein expression analysis or 96-well CellBIND clear bottom black microplates (Corning, Corning, NY, USA) for iRFP reporter assays and grown overnight prior to being transfected using GeneJuice (Merck Millipore) according to the manufacturer's manual. Twenty-four hours after co-transfection, cells were harvested as described above for protein expression analysis or scanned using an Odyssey infrared scanner (LiCor Biosciences). For quantification, plates were scanned at 169 *μ*M resolution, 3.5 mm offset and a low-intensity setting.^23^

### Chromatin-immunoprecipitation

Assays were performed as previously described.^40^ Cells were seeded in a 10-cm plate in DMEM and allowed to grow for 24 h before treatment with CDDP for 24 h.

### Quantification and statistical analysis

Image Studio software (LiCor, V2.1.10) was used to quantify western blots as well as iRFP reporter assays on 96-well plates. The data represent mean values±S.E.M. from at least three independent experiments (*n*=3) unless otherwise noted. All *P* values were obtained using *a t*-test.

## Abbreviations

BPAG1: bullous pemphigoid antigen 1
CDDP: cisplatin
DMEM: Dulbecco's modified Eagle's medium
F-2,6-P_2_: fructose-2,6-bisphosphate
hBS: human p53-binding site
IR: irradiation
iRFP: infrared fluorescent protein
PFK-1: phosphofructokinase-1
PFK-2/FBPase-2: phosphofructokinase-2/fructose-2,6-bisphosphatase
TIFs: tert-immortalised fibroblasts
TIGAR: *TP53*-induced glycolysis and apoptosis regulator
WT: wild type
