# Supplementary material for: p53- and p73-independent activation of TIGAR expression in vivo
Source: Cell Death Dis. 2015 Aug 6;6(8):e1842–. doi: 10.1038/cddis.2015.205 (PMC4558498; doi:10.1038/cddis.2015.205)
Supplement: Supplementary Information [file cddis2015205x1.doc]

**Supplementary Figure 1: Confirmation of TIGAR antibody specificity.** (A) Western blot analysis of TIGAR protein expression in small intestine tissue of wild-type (WT) and TIGAR^-/-^ mice 72 hr after 14 Gy IR. (B) Immunohistochemistry on small intestines from WT and TIGAR^-/-^ animals 72 hr after 14 Gy IR. Scale bar, 20 μm.
